# Markerless gene deletion in *Porphyromonas gingivalis* using a pheS*-based counterselection system

**DOI:** 10.1128/spectrum.01774-26

**Published:** 2026-08-04

**Authors:** Hey-Min Kim, Mary Ellen Davey, Manda Yu

**Affiliations:** 1 Department of Microbiology, ADA Forsyth Institute 1816, Somerville, Massachusetts, USA; Sichuan University, Chengdu, China

**Keywords:** *Porphyromonas gingivalis*, markerless gene deletion, *pheS *counterselection, suicide plasmid, homologous recombination, oral microbiology

## Abstract

*Porphyromonas gingivalis* is an oral pathobiont implicated in periodontitis and several systemic diseases and serves as an important model organism. However, the routine generation of markerless mutants in *P. gingivalis* has remained challenging due to the lack of an efficient counterselection system for the double cross-over approach. Markerless gene deletion is crucial for bacterial genetic manipulations, in particular for generating multiple gene deletions or introducing point mutations. In this study, a counterselection system for *P. gingivalis* was established by placing the pheS* gene under the control of a *P. gingivalis* promoter enabling efficient expression. The construct was delivered to *P. gingivalis* via a suicide plasmid by conjugation. Using PG0719 as a representative target gene, first cross-over recombinants were selected using erythromycin resistance encoded on the suicide plasmid. Cells were then subjected to counterselection in the presence of *p*-chloro-phenylalanine (*p*-Cl-Phe). Retention of pheS* in the genome reduced viability, thereby enriching recombinants that had undergone a second recombination event and loss of the plasmid sequence. Candidate clones were screened by colony PCR analysis to confirm the loss of the gene of interest. A markerless PG0719 mutant was generated and further validated by Sanger sequencing, demonstrating a practical approach for markerless gene deletion in *P. gingivalis* and providing a framework for further genome modifications in the organism.

**IMPORTANCE:**

Although *Porphyromonas gingivalis* is a widely studied model organism, the genetic manipulation of this bacterium has remained limited by the lack of efficient tools for markerless genome editing. Here, we established a counterselection system based on *pheS^∗^* that enables markerless gene deletion in *P. gingivalis*. This approach addresses a technical limitation in the field and provides a practical and broadly applicable framework for advanced genetic manipulation in this important oral pathobiont.

## INTRODUCTION

The human oral cavity harbors a complex microbial community that contributes to both health and disease (1). Among the microbiota, *Porphyromonas gingivalis* is an oral pathobiont associated with chronic inflammation, tissue destruction, and several systemic diseases, including Alzheimer’s disease, cardiovascular disease, and rheumatoid arthritis (2–5). Because of its ability to modulate host immune responses and alter microbial community structure, *P. gingivalis* has become an important model for studying polymicrobial dysbiosis and disease progression (6, 7).

Genetic manipulation of *P. gingivalis* has been successfully used to dissect the molecular mechanisms underlying its virulence, secretion systems, and regulatory pathways (8). However, the current strategies for genetic manipulation in *P. gingivalis* require the use of antibiotic-resistant marker genes (9), as no established protocol has been optimized for generating markerless mutants. In other bacterial systems, a method known as double cross-over recombination is widely used to create markerless mutants (10). In this approach, the first recombination event is selected using an antibiotic resistance marker to facilitate integration of foreign DNA into the genome, while the second recombination event employs a counter-selectable marker to enable the removal of the antibiotic resistance cassette. For example, *sacB* confers sucrose sensitivity and has been widely used for counterselection in other bacterial species (11, 12). Since *P. gingivalis* is asaccharolytic and lacks sugar transporters, a sucrose-based counterselection system cannot be employed. In addition, issues with promoter compatibility and selectable markers create further challenges, so universal double cross-over plasmids have not been directly used to generate markerless mutants in *P. gingivalis*. The lack of a markerless strategy makes multiple gene knockouts particularly challenging due to the limited availability of selectable markers. In addition, accumulation of multiple antibiotic resistance genes within a single bacterial cell may have adverse effects (13). For example, a triple gingipain knockout mutant (KDP136) is available, but it carries three selectable markers (*rgpA2*::Em*^r^ rgpB2*::Tc^r^ *kgp-2*::Cm^r^) (14), and its construction required a complex, multi-step procedure that is difficult to reproduce. Apart from the *sacB*-based approach, mutations in the phenylalanyl-tRNA synthetase α-subunit gene (*pheS*) have been used as counterselection markers in a variety of bacterial species (11, 12). PheS normally functions in charging tRNA^Phe^ with phenylalanine during protein synthesis. Certain mutant PheS variants exhibit altered substrate recognition and can utilize the phenylalanine analog *p*-chloro-phenylalanine (*p*-Cl-Phe) in place of phenylalanine. Subsequent incorporation of *p*-Cl-Phe into newly synthesized proteins results in cellular toxicity, thereby enabling negative selection of cells carrying pheS*. This property has been exploited as a counterselection system for markerless mutagenesis, including in members of the genus *Bacteroides* (15). In this study, a pheS*-based counterselection system was adapted for markerless gene knockout in *P. gingivalis*, and PG0719 in strain W83 was selected as the target. The replacement of PG0719 using an erythromycin resistance gene did not impair growth under normal conditions, suggesting that the deletion of this gene would not impose a substantial selective disadvantage during the second cross-over process. Moreover, this gene is absent in strains such as ATCC 33277, making it a suitable candidate for assessing the feasibility of this approach. PG0719 is predicted to encode a sensor histidine kinase that forms part of a two-component system involved in hemin acquisition and regulation of virulence-associated genes in *P. gingivalis* (16). In addition, coordinated regulation of PG0719 and the downstream response regulator PG0720 highlights the importance of precise genetic manipulation for understanding regulatory networks in this organism (17). Using the pheS*-based counterselection system and this target gene, we aimed to demonstrate the feasibility of markerless gene deletion in *P. gingivalis* and establish a framework for future genetic manipulation in this organism.

## MATERIALS AND METHODS

### Bacterial strains and growth conditions

*P. gingivalis* strains used in this study were derived from the W83 background. *Escherichia coli* strains were used for routine cloning and plasmid propagation. Strains and plasmids used in this study are listed in Table S1.

*P. gingivalis* strains were stored at −80°C and routinely subcultured on trypticase soy agar plates supplemented with 5 µg/mL hemin, 1 µg/mL menadione, and 5% defibrinated sheep blood (Northeast Laboratory Services, Winslow, ME, USA) (BAPHK). Plates were incubated at 37°C for 3–5 days in an anaerobic chamber (Coy Lab Products, Grass Lake, MI, USA) containing an atmosphere of 85% N₂, 10% CO₂, and 5% H₂. For planktonic culture, *P. gingivalis* strains were grown in trypticase soy broth (Becton, Dickinson and Company, Franklin Lakes, NJ, USA) supplemented with hemin and menadione (TSBHK) under the same anaerobic conditions. When required, 10 µg/mL erythromycin was added to the medium for selection. For counterselection, *p*-chloro-phenylalanine (*p*-Cl-Phe) was added to agar medium at the indicated concentrations. *E. coli* strains were grown in Luria–Bertani (LB) broth or on LB agar plates at 37°C. *E. coli* strains harboring plasmids were maintained in media supplemented with 50 µg/mL kanamycin.

### Construction of the pKN-pheS* plasmid

The pKN-pheS* plasmid was derived from pKNOCK-Km (Plasmid #46262, Addgene) (18), a suicide plasmid that does not replicate in *P. gingivalis*. A DNA fragment containing the erythromycin resistance gene (Em) with its native promoter was synthesized by Azenta Life Sciences. The Em cassette, including pUC-GW-Amp-derived sequences, was amplified by PCR and subcloned into pKNOCK-Km using XbaI and KpnI restriction sites. The pUC-GW-Amp-derived sequence contains an origin of replication that enables the propagation of the resulting plasmid in *E. coli* NEB 5α.

A fragment encoding the mutated phenylalanyl-tRNA synthetase allele carrying an A303G substitution (pheS*) from *Bacteroides thetaiotaomicron* under the control of the *P. gingivalis nusG* (PG0389) promoter (263 bp) was synthesized by Azenta Life Sciences and inserted into the plasmid using XbaI and XhoI restriction sites. A SmaI site was introduced at the 5′ end of the *nusG* promoter to facilitate cloning of homologous regions.

### Construction of the pheS*-based PG0719 deletion plasmid

For the deletion of PG0719, approximately 800-bp upstream (U) and 800-bp downstream (D) DNA fragments flanking PG0719 were amplified by PCR using primers designed with the NEBuilder Assembly Tool. The amplified fragments were inserted into the SmaI-digested pKN-pheS* plasmid using the NEBuilder HiFi DNA Assembly Master Mix (NEB). The resulting construct (pKN-pheS*-UD) was verified by PCR and Sanger sequencing. Primer sequences used for plasmid construction are listed in Table S2.

### Selection of single cross-over recombinants and counterselection

The PG0719 deletion plasmid (pKN-pheS*-UD) was introduced into *P. gingivalis* by conjugation as previously described (16). In brief, 10 μg/mL erythromycin was used to select for *P. gingivalis* strains in which the pKN-pheS*-UD plasmid had integrated into the chromosome, while 200 μg/mL gentamicin was used to counterselect the *E. coli* S17-1 donor. Transconjugants were selected on BAPHK agar containing erythromycin and gentamicin under anaerobic conditions. A single erythromycin-resistant transconjugant colony was selected, and single cross-over integration at either of the approximately 800-bp flanking regions of the PG0719 locus was confirmed by PCR using primer pairs spanning the chromosome-plasmid junctions.

To determine optimal counterselection conditions, parent strain W83 and the first cross-over recombinant clones were passaged in non-selective TSBHK medium and plated onto BAPHK containing various concentrations of *p*-Cl-Phe (0, 10, and 15 mM). Growth inhibition was assessed by serial dilution spotting assays. Based on the results, 15 mM *p*-Cl-Phe was used for subsequent experiments. To induce the second cross-over event, first cross-over recombinants were cultured in non-selective TSBHK medium to allow a second recombination event and then plated onto BAPHK containing 15 mM *p*-Cl-Phe.

### Verification of markerless deletion of PG0719

Colonies obtained after counterselection on BAPHK containing 15 mM *p*-Cl-Phe were screened by colony PCR to distinguish PG0719 deletion mutants from wild-type revertants. Verification was performed using primers located in chromosomal regions outside the flanking regions of PG0719. For colony PCR, 50 μL of sterile distilled water was added to each microcentrifuge tube. A portion of each colony was picked using a sterile pipette tip and resuspended thoroughly in sterile distilled water by pipetting. The suspensions were then heated at 97°C for 5 min using a heat block. Subsequently, 0.5 μL of each heated suspension was used as the PCR template in a total reaction volume of 10 μL. PCR amplification was performed using GoTaq Green Master Mix (Promega, Madison, WI, USA) or Phusion High-Fidelity PCR Master Mix (New England Biolabs, Ipswich, MA, USA). PCR products were analyzed by agarose gel electrophoresis, and clones showing the expected amplicon size relative to the parental strain were identified as candidate deletion mutants. Selected amplicons were further subjected to Sanger sequencing using the same external primers to confirm precise deletion of PG0719 and the absence of plasmid-derived sequences at the target locus.

### Erythromycin sensitivity assay for validation of markerless mutant

An erythromycin sensitivity assay was performed to confirm loss of the suicide plasmid-derived erythromycin resistance cassette in the markerless ΔPG0719 mutant. The parent strain W83, the first cross-over recombinant strain carrying pKN-pheS*-UD (W83/pKN-pheS*-UD), and the counterselected ΔPG0719 mutant were streaked onto a non-selective BAPHK plate and a BAPHK plate supplemented with 10 μg/mL erythromycin. Plates were incubated under anaerobic conditions for 3 days. Growth on the non-selective BAPHK plate was used to confirm viability of all strains, whereas growth on the erythromycin-containing plate was used to assess retention of the erythromycin resistance cassette.

### Quantitative RT-PCR analysis

The parent strain W83 and the ΔPG0719 mutant were grown in TSBHK broth to an OD_600_ of approximately 0.5. Total RNA was extracted using the Direct-zol RNA Miniprep kit (Zymo Research) according to the manufacturer’s instructions. cDNA was synthesized from equal amounts of RNA using cDNA EcoDry Premix (Takara Bio USA). Quantitative RT-PCR was performed using gene-specific primers and PowerUp SYBR Green Master Mix (Applied Biosystems, Thermo Fisher Scientific) on a QuantStudio 3 Real-Time PCR System (Applied Biosystems). Transcript levels were normalized to PG0035 (dnaE), encoding the DNA polymerase III alpha subunit.

### Whole-genome sequencing analysis of the ΔPG0719 mutant

Genomic DNA was extracted from the ΔPG0719 mutant using the FastDNA Spin Kit for Soil (MP Biomedicals) according to the manufacturer’s instructions. Briefly, bacterial strains were grown on BAPHK plates and inoculated into TSBHK broth for 24 h. A 2 mL aliquot of overnight culture was harvested by centrifugation prior to DNA extraction. Whole-genome sequencing was performed by Plasmidsaurus (South San Francisco, CA, USA) using Oxford Nanopore Technology with custom analysis and annotation. Sequencing reads were aligned to the *P. gingivalis* W83 reference genome to confirm the intended PG0719 deletion and assess the presence of unintended genomic alterations. Genome comparisons and visualization were performed using progressiveMauve (19).

## RESULTS

### Construction of the pKN-pheS*-UD suicide plasmid for *P. gingivalis*

**Fig 1:**
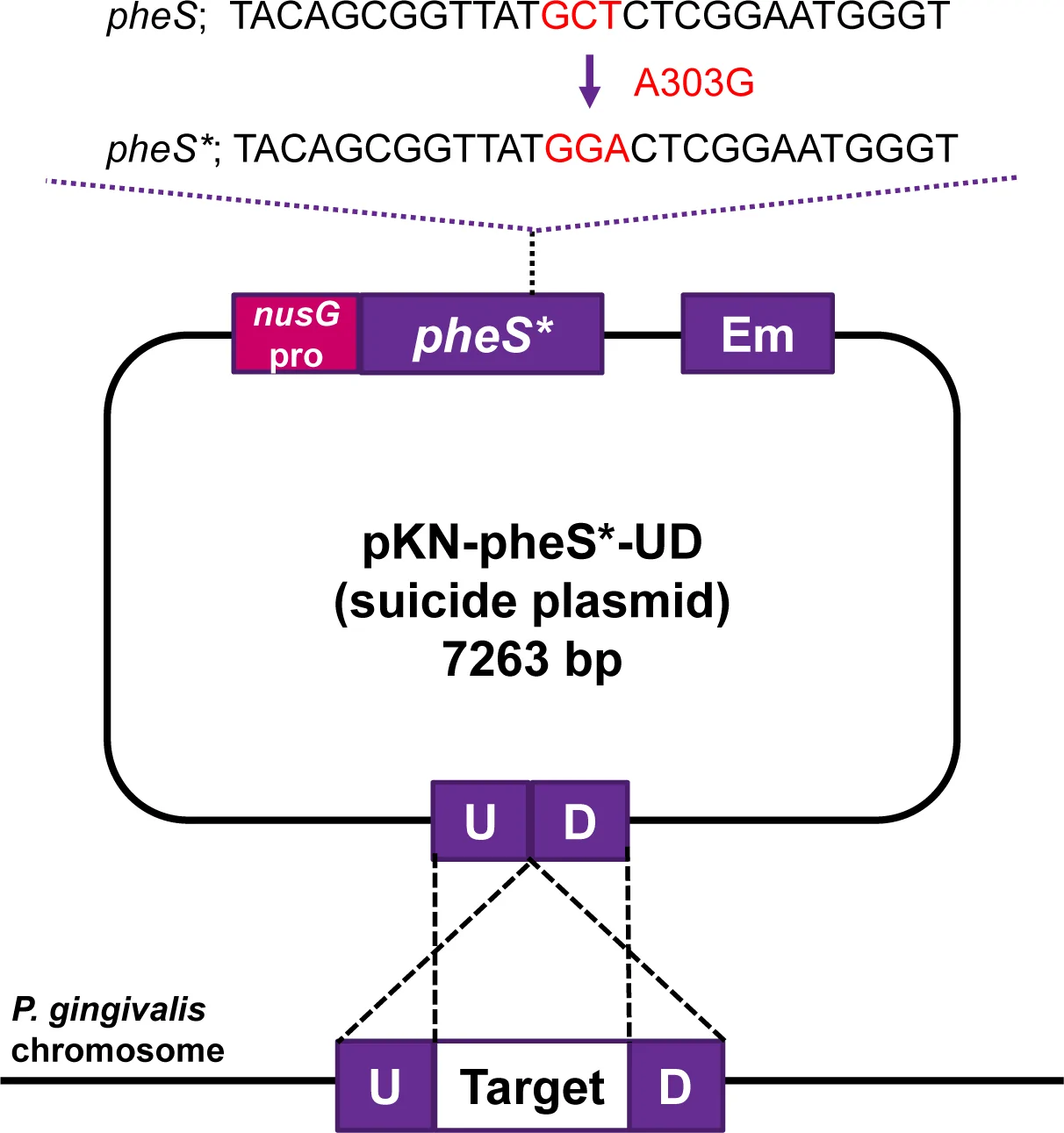
Construction of the pKN-pheS*-UD suicide plasmid for *P. gingivalis*. Schematic representation of the pKN-pheS*-UD plasmid (7,263 bp). The plasmid carries a mutated *pheS* allele (pheS*, A303G) under the control of the *P. gingivalis nusG* promoter, together with an erythromycin resistance cassette (Em). Approximately 800-bp upstream (U) and downstream (D) homologous regions flanking the target locus were inserted for homologous recombination. The pheS* mutation confers sensitivity to *p*-chloro-phenylalanine (*p*-Cl-Phe), enabling counterselection.

To establish a markerless gene deletion system in *P. gingivalis*, we constructed the suicide plasmid pKN-pheS*-UD (7,263 bp), which carries a mutated *pheS* allele (pheS*) under the control of the *P. gingivalis nusG* promoter, together with an erythromycin resistance cassette (Em). The A303G substitution was introduced into *pheS* to confer sensitivity to *p*-chloro-phenylalanine (*p*-Cl-Phe), enabling counterselection (Fig. 1). The plasmid also includes approximately 800-bp upstream (U) and 800-bp downstream (D) homologous regions flanking the target gene locus (PG0719), allowing for site-specific integration into the chromosome via homologous recombination. The overall design enables both positive selection (erythromycin resistance) and negative selection (pheS*-mediated sensitivity). A schematic overview of the pheS*-based counterselection system used in this study is provided in Fig. S1.

### Single cross-over integration of the suicide plasmid

**Fig 2:**
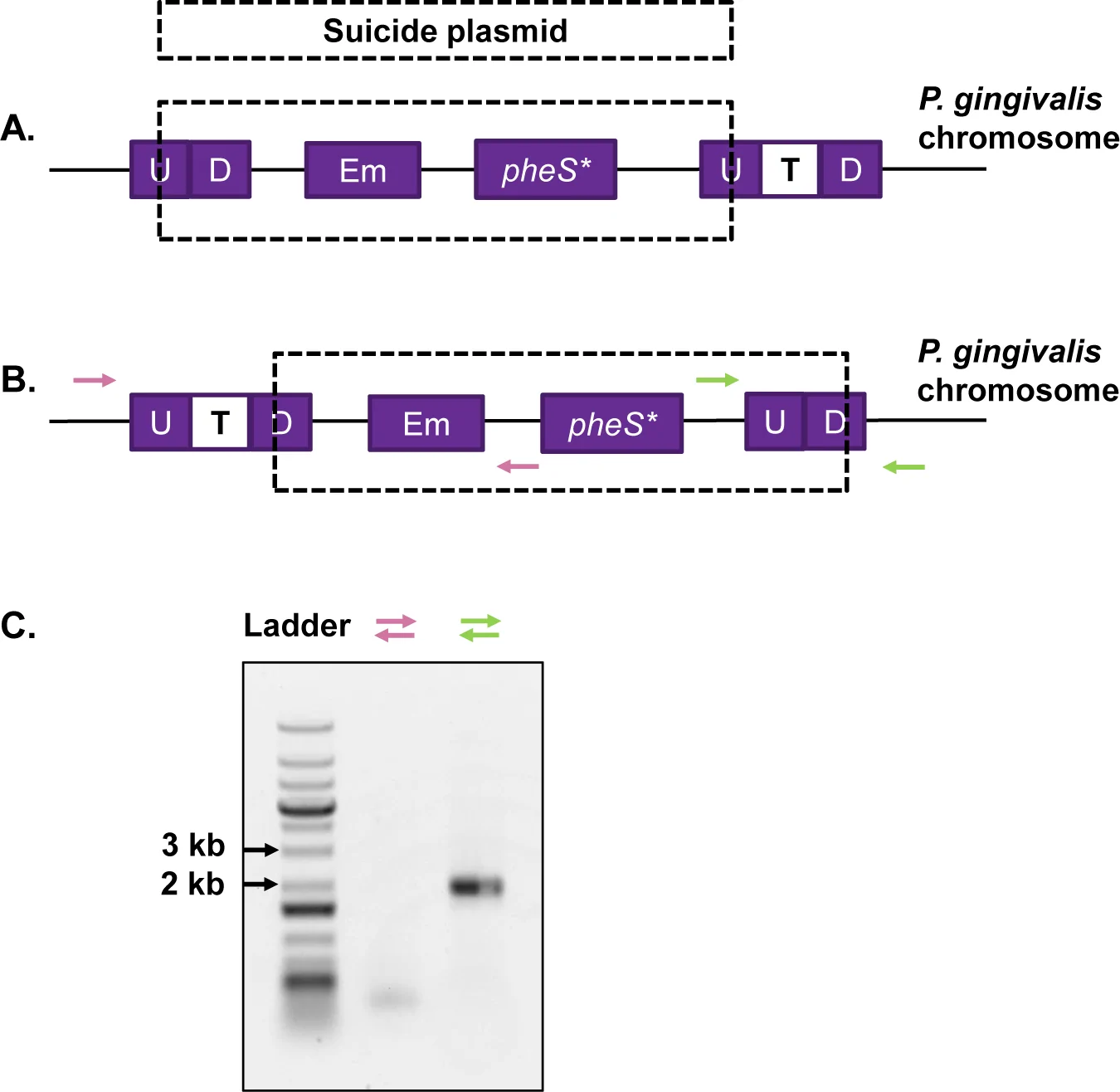
Confirmation of first cross-over recombinants. (**A**) Schematic representation of single cross-over integration via homologous recombination through the upstream homologous region (U). (**B**) Schematic representation of single cross-over integration via homologous recombination at the downstream homologous region (D). In both cases, integration results in insertion of the entire plasmid, including the erythromycin resistance cassette (Em) and pheS*, into the chromosome. (**C**) PCR verification of a representative erythromycin-resistant integrant. Primer pairs were designed to distinguish the two possible integration configurations. The observed PCR product corresponds to the downstream cross-over configuration shown in panel **B**, confirming successful chromosomal integration of the suicide plasmid.

To examine chromosomal integration of the pKN-pheS*-UD suicide plasmid, the expected first cross-over structures were first defined based on recombination occurring through either the upstream or downstream homologous region. As shown in Fig. 2A, homologous recombination through the upstream homologous region would generate an integrated structure in which the plasmid sequence is inserted adjacent to the intact target locus. Alternatively, recombination through the downstream homologous region would yield the configuration shown in Fig. 2B. In both cases, the entire suicide plasmid, including the erythromycin resistance cassette and pheS*, is incorporated into the chromosome, thereby generating an erythromycin-resistant recombinant.

To determine which integration pattern had occurred in the selected erythromycin-resistant clone, PCR analysis was performed using diagnostic primer pairs designed to distinguish the two possible insertions. As shown in Fig. 2C, the PCR product obtained was consistent with the structure predicted for recombination through the downstream homologous region, confirming that the analyzed isolate had undergone single cross-over integration in the configuration illustrated in Fig. 2B. These results verify successful chromosomal integration of the suicide plasmid and demonstrate that erythromycin selection enabled recovery of first cross-over recombinants in *P. gingivalis*.

### *p*-Cl-Phe-mediated counterselection

**Fig 3:**
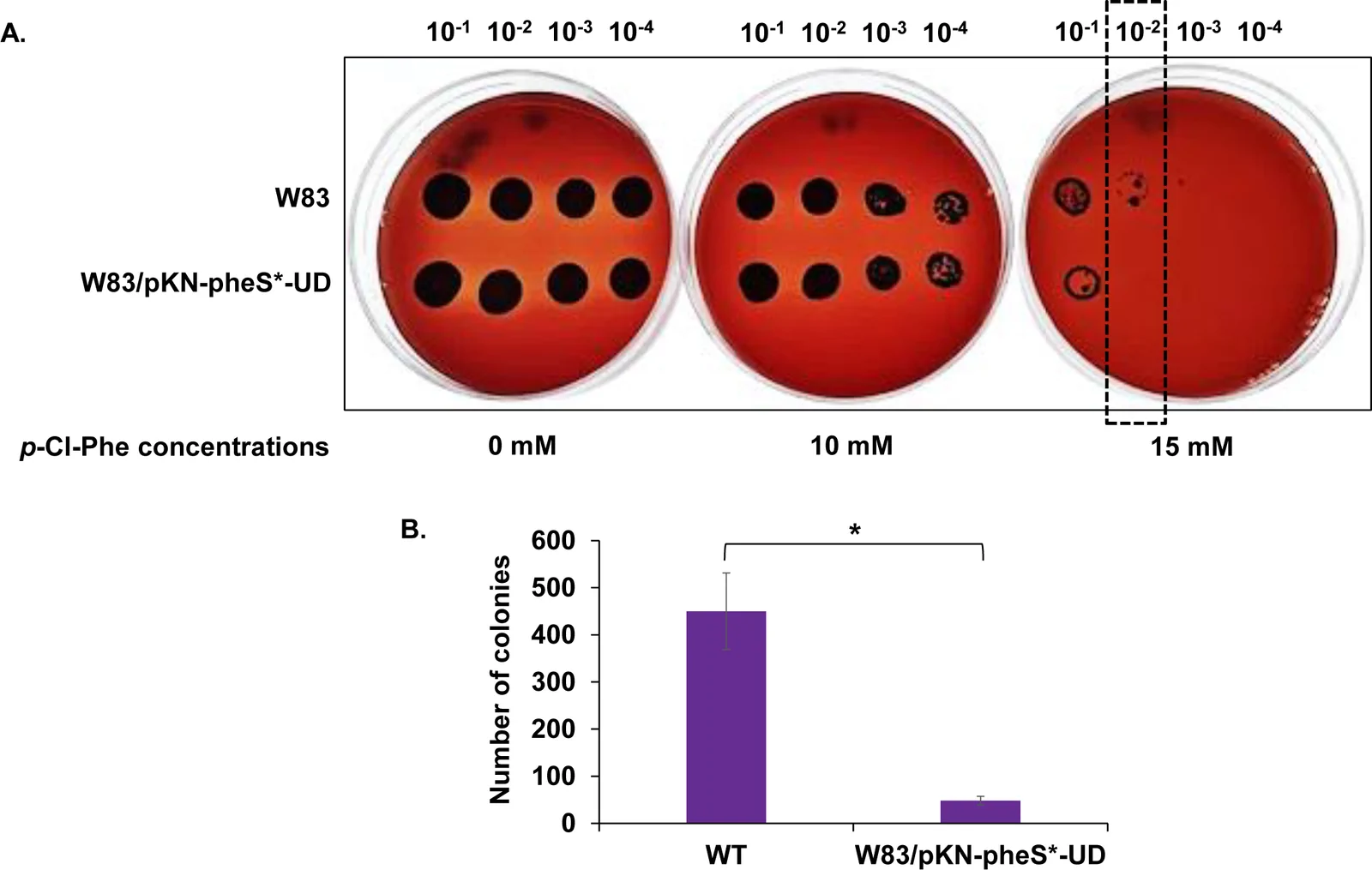
Determination of *p*-Cl-Phe counterselection conditions. (**A**) Serial dilution spotting assay showing growth of *P. gingivalis* W83 and W83/pKN-pheS*-UD strains on agar plates containing increasing concentrations of *p*-chloro-phenylalanine (*p*-Cl-Phe). Both strains exhibited similar growth at 0 mM and 10 mM *p*-Cl-Phe. At 15 mM *p*-Cl-Phe, the pheS*-containing strain exhibited reduced growth compared to parent strain W83. Plates were photographed after 7 days of incubation. (**B**) Quantification of colony formation on 15 mM *p*-Cl-Phe agar plates. Equal volumes (100 μL) of a 10^−2^ dilution from each culture were spread onto agar plates, and colonies were counted after incubation. * Data are means ± SD from three replicates by Student’s *t* test with a significant *P* value of  <  0.05.

To determine effective conditions for counterselection, the sensitivity of *P. gingivalis* strains to *p*-chloro-phenylalanine (*p*-Cl-Phe) was assessed using a serial dilution spotting assay (Fig. 3A). The parent strain W83 was included as a control to evaluate background sensitivity to *p*-Cl-Phe. In the absence of *p*-Cl-Phe, both the parent strain W83 and the pheS*-containing strain exhibited comparable growth across all dilutions, indicating that expression of pheS* does not affect viability under non-selective conditions. Notably, at 15 mM *p*-Cl-Phe, the pheS*-containing strain was more sensitive than the parent strain W83, with no detectable growth observed at the 10⁻² dilution. To further quantify the difference in *p*-Cl-Phe sensitivity, equal volumes of a 10⁻² dilution from each culture were spread onto 15 mM *p*-Cl-Phe agar plates, and colony formation was assessed (Fig. 3B). Consistent with the spotting assay, the pheS*-containing strain produced significantly fewer colonies than the parent strain W83 (*P* < 0.05), confirming increased sensitivity to *p*-Cl-Phe. Together, these results indicate that 15 mM *p*-Cl-Phe provides effective counterselection for selectively eliminating cells harboring pheS*.

### Screening for markerless deletion of PG0719

**Fig 4:**
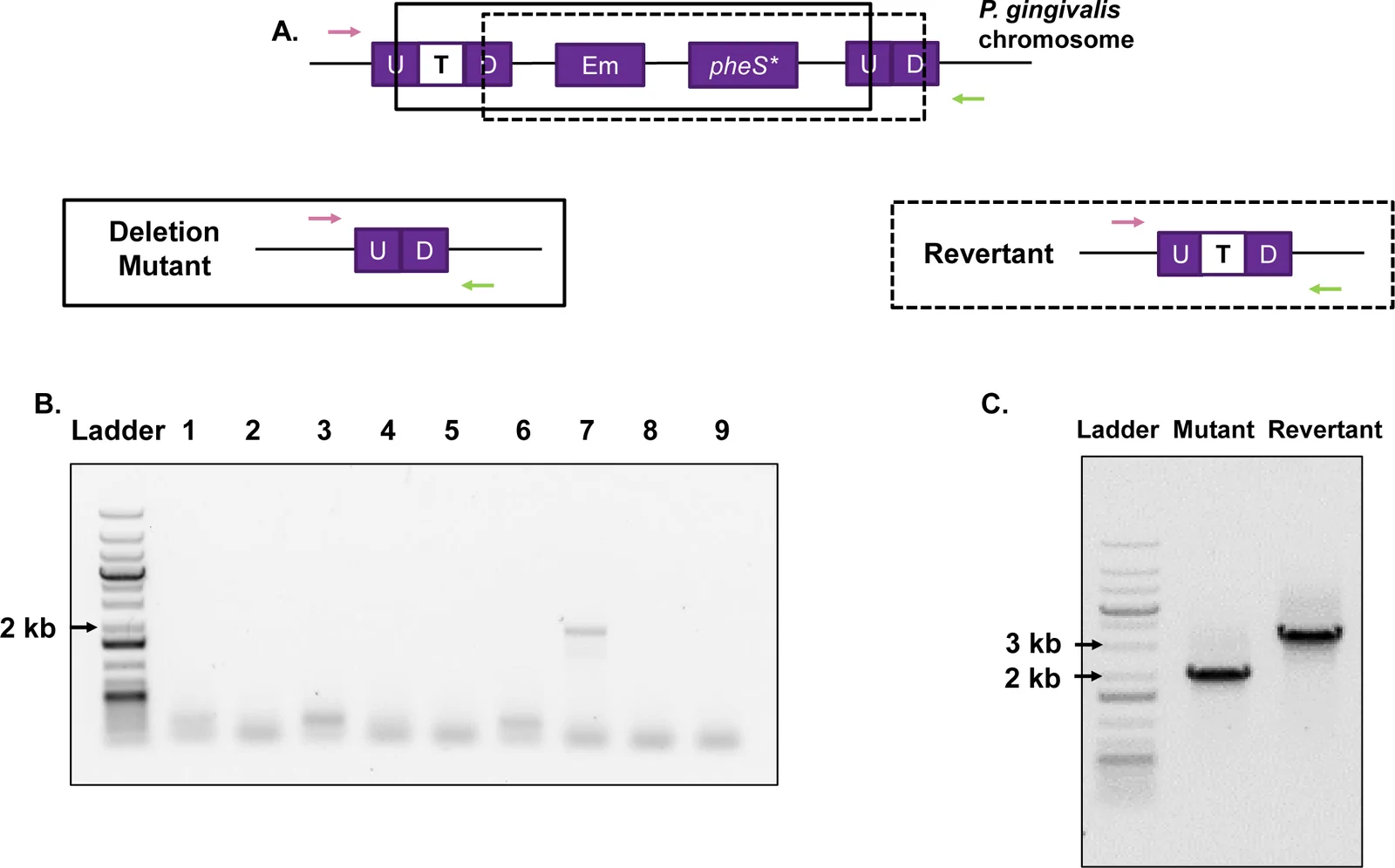
Screening of markerless deletion of PG0719. (**A**) Schematic representation of markerless deletion of PG0719 following double cross-over recombination. U and D indicate the upstream and downstream homologous regions, respectively. The suicide plasmid was first integrated into the chromosome by a single cross-over event at the downstream homologous region. A second cross-over at the upstream homologous region excised the integrated plasmid together with the target gene (T), generating the markerless deletion allele (U–D). In contrast, recombination at the downstream homologous region restored the wild-type allele (U–T–D). Arrows indicate the positions of the external screening primers used for PCR analysis. (**B**) PCR products expected from the external primer set in colony PCR assays performed with Taq DNA polymerase and a 2 min elongation time. Successful markerless deletion produces a smaller amplicon corresponding to the deletion allele (U–D), whereas the wild-type allele (U–T–D) is expected to generate a larger PCR product due to the presence of PG0719 (1,284 bp). (**C**) PCR amplification of colony 7 and colony 8 from panel B using Phusion High-Fidelity PCR Master Mix and the same external primer set.

To screen for markerless deletion of PG0719 following *p*-Cl-Phe counterselection, candidate colonies were analyzed by colony PCR using primers located outside the homologous regions (Fig. 4A). This PCR assay distinguishes the deletion allele (U–D) from the wild-type revertant allele (U–T–D) based on PCR product size, with the deletion allele producing a smaller amplicon due to removal of PG0719 (1,284 bp). Initial colony PCR screening was performed using Taq DNA polymerase with a 2 min elongation time (Fig. 4B). Among the nine screened colonies, only colony 7 produced an approximately 2-kb PCR product, consistent with the expected size of the PG0719 deletion allele. The absence of visible PCR products in the remaining colonies was likely due to inefficient amplification of the larger wild-type revertant allele under the 2 min colony PCR conditions. To further confirm the PCR genotypes, colony 7 and colony 8 were reanalyzed using Phusion High-Fidelity PCR Master Mix and the same external primer set (Fig. 4C). Colony 7 generated the smaller PCR product expected for the PG0719 deletion allele, whereas colony 8 generated the larger PCR product expected for the wild-type revertant allele. These results demonstrate that *p*-Cl-Phe counterselection enables recovery of markerless recombinants and that deletion mutants can be distinguished from wild-type revertants by PCR.

### Validation of markerless deletion of PG0719

**Fig 5:**
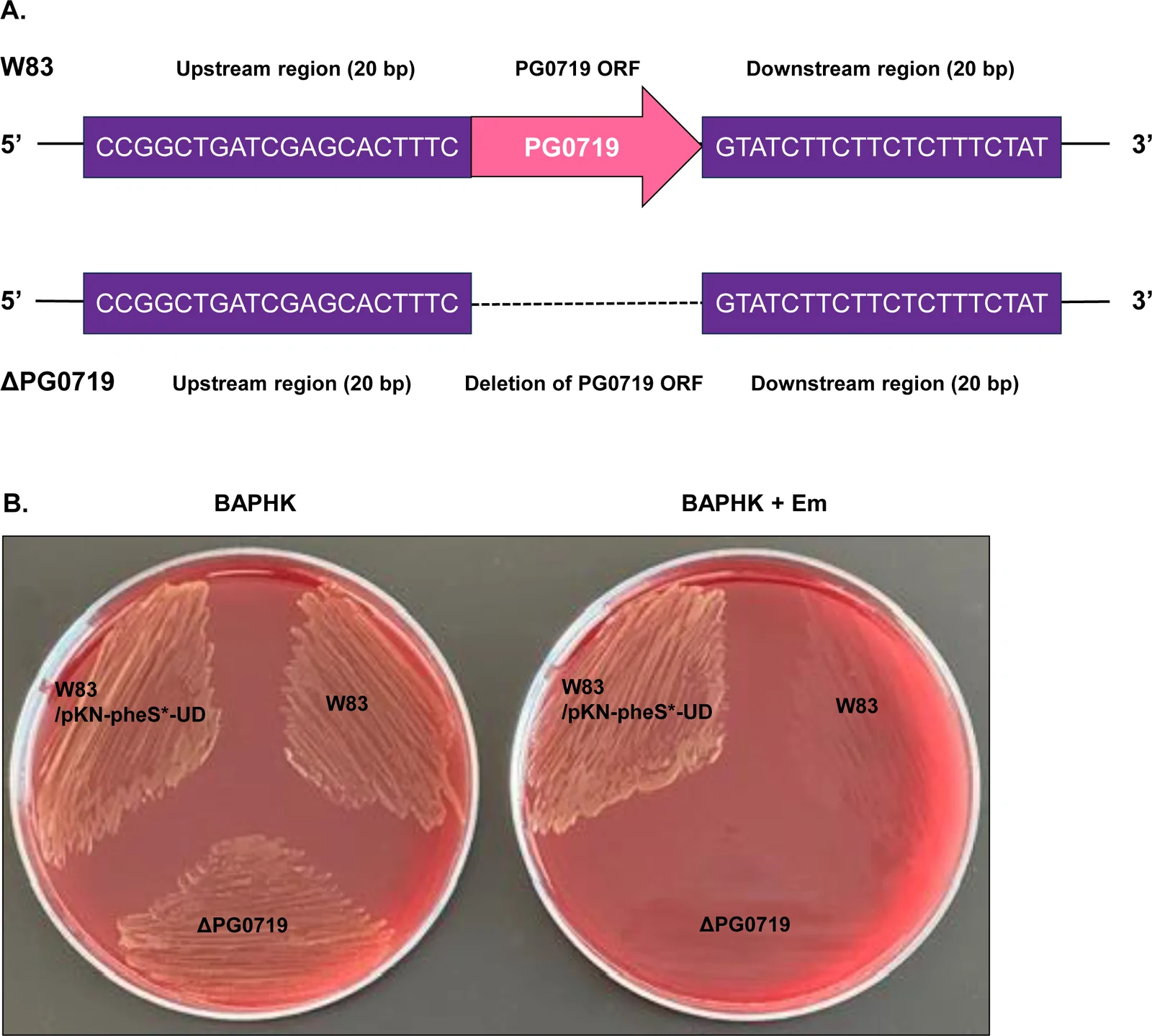
Validation of markerless deletion of PG0719. (**A**) Sanger sequencing of PCR amplicons spanning the PG0719 locus was performed using external primers to confirm precise deletion of the PG0719 open reading frame (ORF) in the markerless ΔPG0719 mutant and the absence of plasmid-derived sequences at the target locus. (**B**) The parent strain W83, the first cross-over recombinant strain (W83/pKN-pheS*-UD), and the ΔPG0719 mutant were compared for growth on BAPHK plates in the absence or presence of 10 μg/mL erythromycin. Plates were photographed after 3 days of incubation.

To further validate the markerless deletion of PG0719, PCR amplicons spanning the target locus were subjected to Sanger sequencing using external primers located outside the homologous regions (Fig. 5A). Sequence analysis confirmed precise deletion of the PG0719 open reading frame (ORF) in the ΔPG0719 mutant, resulting in direct fusion of the upstream and downstream flanking regions. No plasmid-derived sequences were detected in the ΔPG0719 mutant sequence, confirming successful excision of the suicide plasmid following counterselection. These results confirmed precise deletion of PG0719 without residual plasmid integration at the target locus.

In addition, erythromycin resistance was assessed following counterselection (Fig. 5B). On non-selective BAPHK plates, the parent strain W83, the first cross-over recombinant strain (W83/pKN-pheS*-UD), and the ΔPG0719 mutant all exhibited comparable growth. In contrast, on BAPHK plates supplemented with erythromycin, only the W83/pKN-pheS*-UD strain retained growth, whereas both W83 and the ΔPG0719 mutant strains failed to grow. The parent strain served as a negative control for erythromycin resistance, confirming that the ΔPG0719 mutant had lost the pKN-pheS*-derived erythromycin resistance cassette.

To further confirm loss of PG0719 expression, quantitative RT-PCR analysis was performed using RNA isolated from mid-log phase cultures. As shown in Fig. S2, PG0719 transcript levels were undetectable in the ΔPG0719 mutant compared to the parent strain W83, confirming loss of PG0719 mRNA following the deletion of the target gene. Whole-genome sequencing was subsequently performed to verify the deletion of the target gene and assess the presence of unintended genomic alterations. Sequence analysis confirmed the expected PG0719 deletion and did not identify additional mutations associated with the deletion process (Fig. S3). Together, these analyses confirmed successful generation of a markerless ΔPG0719 mutant without retention of erythromycin resistance or unintended genomic alterations, making it feasible for additional gene deletions.

## DISCUSSION

In this study, we adapted a pheS*-based counterselection system established in *Bacteroides* to enable markerless gene deletion in *P. gingivalis*. Placement of the pheS* gene under the control of a *P. gingivalis*-native promoter enabled *p*-Cl-Phe-based counterselection during the second cross-over process. To our knowledge, this strategy has not previously been reported in *P. gingivalis*. The promoter of the *nusG* gene (~263 bp) was selected because it is located adjacent to several highly expressed ribosomal protein genes (i.e., 50S ribosomal protein genes PG0390–PG0393). In addition, its relatively short length minimizes the amount of homologous sequence shared between the suicide plasmid and the chromosome, thereby reducing the potential for unintended recombination events (20). A key requirement for successful implementation of this strategy was the functional compatibility of the heterologous pheS* variant from *Bacteroides thetaiotaomicron* in *P. gingivalis. p*-Cl-Phe was applied at 15 mM in BAPHK, a concentration previously reported for counterselection in *Bacteroides* species. Under these conditions, *p*-Cl-Phe effectively distinguished the parent strain W83 from the first cross-over recombinant (Fig. 3). However, compared to *Bacteroides* species, the results were less clear-cut, likely due to the use of BAPHK medium without phenylalanine depletion. To address this, colonies initially selected on BAPHK plates containing 15 mM *p*-Cl-Phe can be re-streaked onto fresh BAPHK plates supplemented with the same concentration (15 mM) of *p*-Cl-Phe to confirm sustained growth under counterselection pressure. In addition, the isolation of single colonies is important. An alternative counterselection-based system based on thymidine kinase (*tdk*) has also been developed to improve the efficiency of markerless gene deletion in *Bacteroides* species (21, 22). In this system, a *tdk* deletion strain is used as the parental background, and a suicide plasmid carrying a functional copy of *tdk* is introduced to facilitate homologous recombination. Following integration, cells retaining the suicide plasmid become sensitive to toxic nucleoside analogs, as *tdk* converts these compounds into lethal intermediates. Consequently, counterselection enables the efficient elimination of suicide plasmid intermediates and enriches for recombination events that have resolved the integrated construct. Compared to pheS*-based counterselection, which relies on sensitivity to *p*-Cl-Phe, the *tdk* system offers an alternative strategy that may reduce background associated with suicide plasmid retention.

PG0719 was used as a representative target locus to validate the feasibility of the markerless gene deletion system as there was no strong growth phenotype in the Em-carrying ΔPG0719 mutant. Detailed phenotypic characterization of the ΔPG0719 mutant is currently ongoing, while the cognate response regulator PG0720 has previously been shown to play a critical role in the regulation of K-antigen capsule synthesis and surface-associated phenotypes in *P. gingivalis* (17). This is particularly relevant for virulence-associated pathways that involve multiple genetic loci and layers of regulation. For example, capsule biosynthesis in *P. gingivalis* is encoded by distinct genomic loci (e.g., PG0106–PG0120 and PG1135–PG1142) and exhibits substantial genetic variation across strains (23). In addition, transcription of these loci can be modulated by DNA-binding proteins such as HU and associated regulatory elements (24). Furthermore, major virulence factors such as gingipains (RgpA, RgpB, and Kgp) are multifunctional cysteine proteases that contribute to host interaction, immune modulation, and tissue destruction (25–27). Their activity also requires coordinated expression, processing, and assembly. Together, these features highlight the need for flexible and precise genetic tools to dissect complex virulence mechanisms in *P. gingivalis*.

A major limitation of the double cross-over strategy is that it cannot be used to delete essential genes. Alternatively, conditional gene expression systems, including inducible promoters (e.g., Tet- or Lac-based systems) (28) and CRISPR interference (CRISPRi) (29), enable modulation of gene expression without complete loss of function. These approaches allow functional interrogation of essential loci while maintaining viability. In parallel, quantitative approaches such as transposon sequencing (TnSeq/InSeq) (30) and barcoded mutant libraries (31) have been used in model gut *Bacteroides* systems under defined experimental conditions. These approaches enable genome-wide, quantitative assessment of mutant fitness, allowing systematic evaluation of how individual genes contribute to bacterial growth under specific conditions. TnSeq has already been established in *P. gingivalis* (32), and similar high-throughput approaches may further facilitate the study of essential genes and condition-specific fitness determinants.

In summary, a pheS*-based counterselection strategy was established that enables markerless gene deletion in *P. gingivalis*. This work expands the genetic toolkit available for this model organism, facilitating more complex genetic manipulations such as multigene knockouts. This strategy is expected to support genetic studies of virulence, regulation, and host–microbe interactions in *P. gingivalis*.

## Data Availability

The genome generated and analyzed in this study has been deposited in the NCBI repository under BioProject accession numbers PRJNA1480070. All other data supporting the findings of this study are available from the corresponding author upon reasonable request.

## SUPPLEMENTAL MATERIAL

Tables S1 and S2 and Figures S1, S2, and S3 spectrum.01774-26-s0001.pdf Supplemental Information

ASM does not own the copyrights to Supplemental Material that may be linked to, or accessed through, an article. The authors have granted ASM a non-exclusive, world-wide license to publish the Supplemental Material files. Please contact the corresponding author directly for reuse.
