# Supplementary material for: Markerless gene deletion in Porphyromonas gingivalis using a pheS*-based counterselection system
Source: Microbiol Spectr. 2026 Aug 4;14(9):e01774-26. doi: 10.1128/spectrum.01774-26 (PMC13532207; doi:10.1128/spectrum.01774-26)
Supplement: Supplementary file 1 [file spectrum.01774-26-s0001.pdf]

Supplemental Information

Table S1. Strains and plasmids used in this study

Strain (relevant genotype)	Source or reference
***P. gingivalis* strains**	
W83	Christian Mouton, Laval University, Quebec City, Canada
W83/pKN-pheS*-UD	This study
ΔPG0719 in strain W83	This study
***E. coli* strain**	
NEB 5α	NEB
S17-1	Matsumoto-Mashimo et al, 2004
**Plasmids**	
pKNOCK-Km	Addgene (Plasmid #46262)
pKN-pheS*	This study
pKN-pheS*-UD	This study

Reference:

Matsumoto-Mashimo C, Guerout AM, Mazel D. 2004. A new family of conditional replicating plasmids and their cognate *Escherichia coli* host strains. Res Microbiol 155:455-61.

Table S2. Primers used in this study

Name	Sequence (5'-3')	Purpose
pKN-seq-FW	AGAAGCCCTTAGAGCCTCTCAAAGC	Sequencing of pKN-pheS*
pKN-seq-RV	CTGTTTGTCAGCTCGGATCTGGTAGG	Sequencing of pKN-pheS*
PG0719_UP_FW	CCGCTCTAGAACTAGTGGATCCCCCGTAAAATATCTGCACATCCC	Deletion of ΔPG0719
PG0719_UP_RV	ATCGAGCACTTTCGTATCTTCTTCTCTTTCTATGAG	Deletion of ΔPG0719
PG0719_DW_FW	AGAAGAAGATACGAAAGTGCTCGATCAGCC	Deletion of ΔPG0719
PG0719_DW_RV	TGAGAGACTCGAACTCGGTACCCCCTAACGCCTTCGTTCATGAATTC	Deletion of ΔPG0719
PG0719_seq_FW	TGCGCCCGAACAAGAGAAGG	Sequencing of ΔPG0719
PG0719_seq_RV	ACTTCCTGGTCTCCCTTTTC	Sequencing of ΔPG0719
PG0035_FW	GATCCGTATCATCCCAGACG	Forward RT-PCR primer for PG0035
PG0035_RV	AAAGCTCCTTGTCGATACGC	Reverse RT-PCR primer for PG0035
PG0719_FW	GCCACTGTATCGACTGATCG	Forward RT-PCR primer for PG0719
PG0719_RV	CTTTCTGCTGCTCGAAAACC	Reverse RT-PCR primer for PG0719

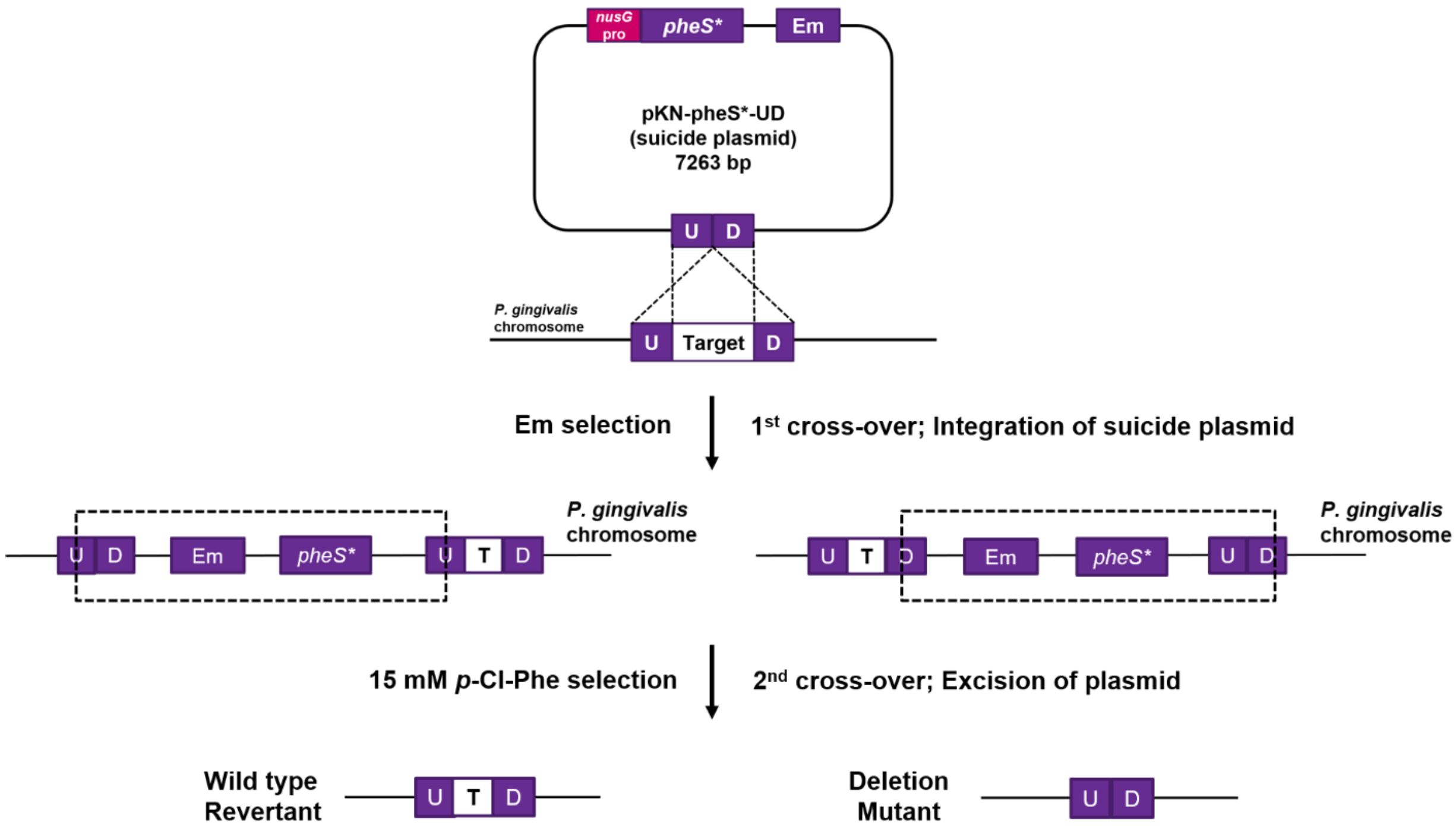

Figure S1. Schematic overview of the *pheS-based counterselection system for markerless gene deletion in *P. gingivalis*.**

The suicide plasmid pKN-pheS* contains the *pheS** gene under the control of the *P. gingivalis nusG* promoter and an erythromycin resistance marker (Em). Upstream (U) and downstream (D) homologous regions flanking the target gene (T) were cloned into the plasmid to facilitate homologous recombination. Following conjugation, erythromycin-resistant single-crossover integrants were selected. A second homologous recombination event resulted in either reversion to the wild-type genotype or deletion of the target gene. Counterselection on *p*-chloro-phenylalanine (*p*-Cl-Phe) eliminated cells retaining the *pheS** gene, enabling the isolation of markerless gene deletion mutants.

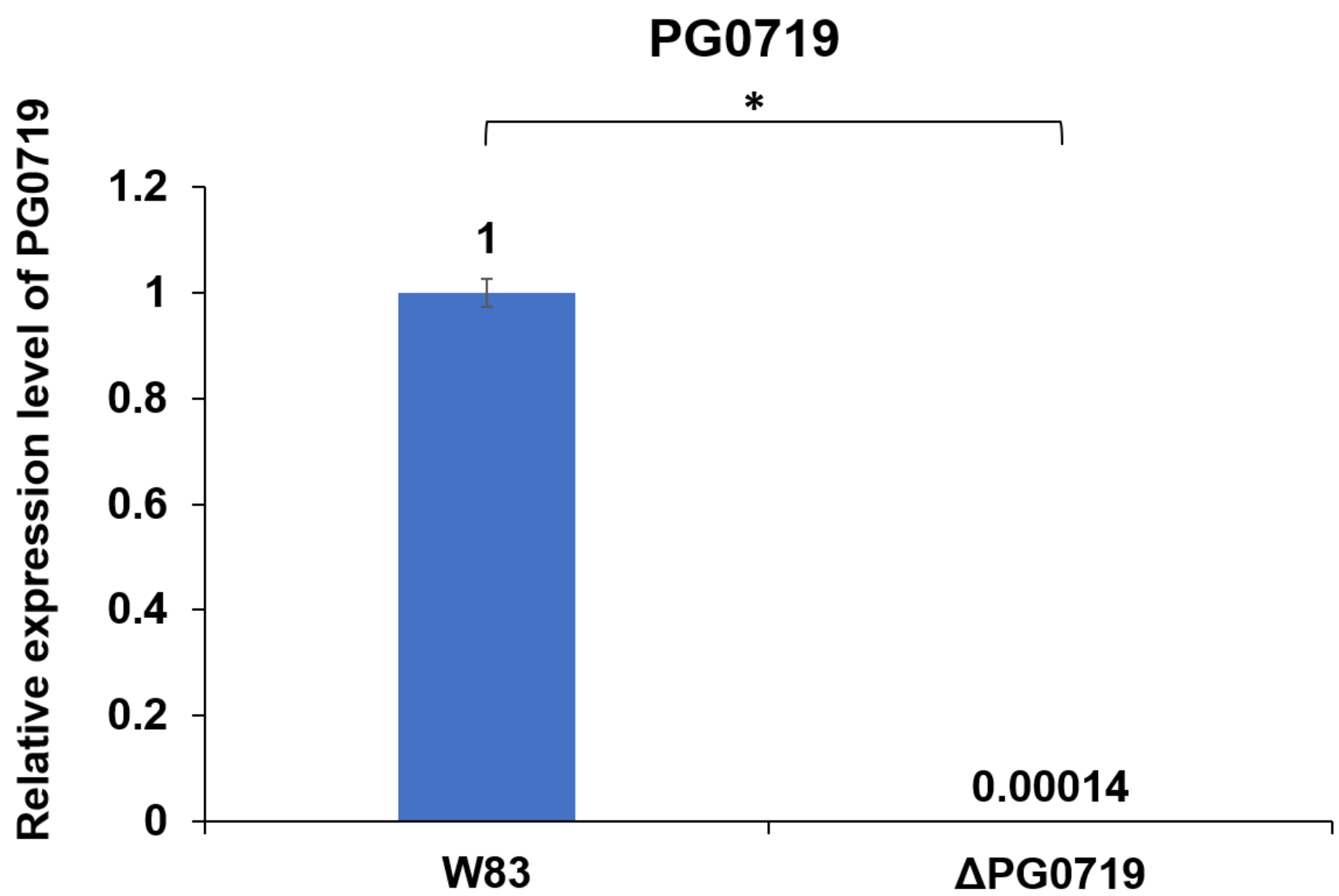

Figure S2. Quantitative RT-PCR analysis of PG0719 expression in the ΔPG0719 mutant.

Relative transcript levels of PG0719 were determined by quantitative RT-PCR in the parent strain W83 and the ΔPG0719 mutant. Expression levels were normalized to PG0035 (dnaE), encoding the DNA polymerase III alpha subunit, and are presented relative to W83, which was set to 1. Data represent the mean ± SD from three replicates. PG0719 transcript levels were undetectable in the ΔPG0719 mutant compared to W83. * Data are means ± SD from three replicates by Student's t test with a significant *P* value of < 0.05.

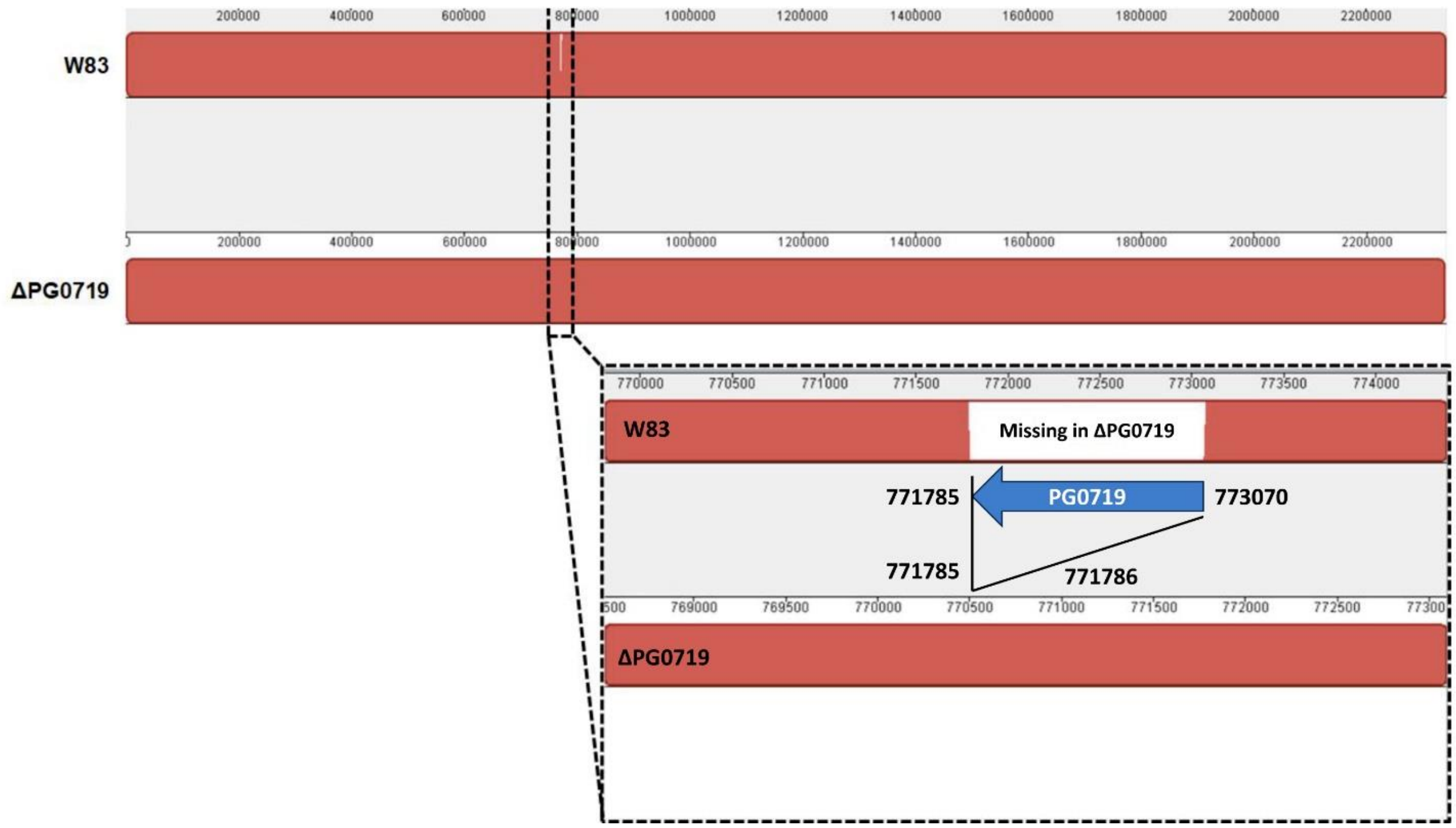

Figure S3. Whole-genome sequencing analysis of the ΔPG0719 mutant.
Whole-genome sequencing was performed on the ΔPG0719 mutant. Genome alignment against the W83 reference genome confirmed the expected deletion at the PG0719 locus. The upper panel shows the genome-wide alignment between the ΔPG0719 mutant and the W83 reference genome. The lower panel shows an enlarged view of the region flanking PG0719, demonstrating the absence of the PG0719 open reading frame (Position: 771786-773069 bp) in the ΔPG0719 mutant. No additional large-scale genomic rearrangements were detected.
